# Safety, tolerability, and preliminary activity of IMGN529, a CD37-targeted antibody-drug conjugate, in patients with relapsed or refractory B-cell non-Hodgkin lymphoma: a dose-escalation, phase I study

**DOI:** 10.1007/s10637-018-0570-4

**Published:** 2018-02-17

**Authors:** Anastasios Stathis, Ian W. Flinn, Sumit Madan, Kami Maddocks, Arnold Freedman, Steven Weitman, Emanuele Zucca, Mihaela C. Munteanu, M. Lia Palomba

**Affiliations:** 1 Oncology Institute of Southern Switzerland, Ospedale San Giovanni, Bellinzona, Switzerland; 2 Sarah Cannon Research Institute, Nashville, TN USA; 3 Institute for Drug Development, San Antonio, TX USA; 4 Ohio State University, Columbus, OH USA; 5 Dana Farber Cancer Institute, Boston, MA USA; 6 ImmunoGen Inc., Waltham, MA USA; 7 Memorial Sloan Kettering Cancer Center, New York, NY USA

**Keywords:** Antibody-drug conjugate, CD37, IMGN529, Non-Hodgkin lymphoma, Phase I

## Abstract

*Background* CD37 is expressed on B-cell lymphoid malignancies, thus making it an attractive candidate for targeted therapy in non-Hodgkin lymphoma (NHL). IMGN529 is an antibody-drug conjugate comprising a CD37-binding antibody linked to the maytansinoid DM1, a potent anti-mitotic agent. *Methods* This first-in-human, phase 1 trial recruited adult patients with relapsed or refractory B-cell NHL. The primary objective was to determine the maximum tolerated dose (MTD) and recommended phase 2 dose. Secondary objectives were to evaluate safety, pharmacokinetics, and preliminary clinical activity. IMGN529 was administered intravenously once every 3 weeks, and dosed using a conventional 3 + 3 dose-escalation design. *Results* Forty-nine patients were treated at doses escalating from 0.1 to 1.8 mg/kg. Dose limiting toxicities occurred in eight patients and included peripheral neuropathy, febrile neutropenia, neutropenia, and thrombocytopenia. The most frequent treatment-emergent adverse events were fatigue (39%), neutropenia, pyrexia, and thrombocytopenia (each 37%). Adverse events led to treatment discontinuation in 10 patients (20%). Eight patients (16%) had treatment-related serious adverse events, the most common being grade 3 febrile neutropenia. The MTD (with growth factor support) was 1.4 mg/kg every 3 weeks. IMGN529 plasma exposure increased monotonically with dose and was consistent with target-mediated drug disposition. Five (13%) of 39 response-evaluable patients achieved an objective response (one complete response and four partial responses), four of which occurred in the subgroup of patients with diffuse large B-cell lymphoma. *Conclusions* The manageable safety profile of IMGN529 and preliminary evidence of activity, particularly in DLBCL patients, support the continued development of this novel CD37-targeting agent.

**Electronic supplementary material:**

The online version of this article (10.1007/s10637-018-0570-4) contains supplementary material, which is available to authorized users.

## Introduction

Non-Hodgkin lymphoma (NHL) represents a heterogeneous collection of distinct malignancies of the lymphatic system, each associated with their own pathological features and clinical outcomes [1]. Approximately 85% of NHL diagnoses are of B-cell origin; the most frequent histological subtypes include diffuse large B-cell lymphoma (DLBCL), follicular lymphoma (FL), marginal zone lymphoma (MZL), and mantle cell lymphoma (MCL). Since its introduction into B-cell lymphoma management two decades ago, the anti-CD20 antibody rituximab has improved survival outcomes for patients with DLBCL, FL, and MCL, as well as in other subsets [2]. Indeed, the clinical success of rituximab has served as a paradigm for tailored therapeutic approaches in the treatment of B-cell malignancies and targeting of alternative cell surface antigens (e.g.*,* CD19, CD30) continues to be an actively pursued therapeutic area [2]. An urgent need for improved therapeutic options still exists, particularly for patients with aggressive-histology lymphomas (such as DLBCL) in the refractory and/or relapsed settings, as outcomes for these individuals remain poor [3].

One promising target is the tetraspanin CD37, a transmembrane protein whose exact physiological function(s) are yet to be defined, although there is evidence to suggest it is involved in immune cell proliferation and survival [4, 5]. In normal tissues, CD37 shows a restricted distribution pattern with expression limited to lymphoid tissues - most frequently on the surface of B-cells from the pre-B through the peripheral mature stages of differentiation but absent on early progenitor and terminally differentiated plasma cells [6, 7]. Importantly, CD37 is highly expressed on malignant B-cells, including most subtypes of NHL [8, 9]. This differential expression profile identified CD37 as a candidate for the development of novel therapeutics. A limited number of CD37-targeting approaches have been explored to date, including radioimmunotherapy with a radiolabeled anti-CD37 antibody (^131^I–MB-1) [10, 11], a CD37-binding small immunopharmaceutical protein (TRU-016) [12], and a Fc-engineered antibody (BI836826) [13], with the latter two agents both exhibiting antibody-dependent cell-mediated cytotoxicity (ADCC) and apoptosis-inducing abilities.

Antibody-drug conjugate (ADC) technology provides targeted delivery of cytotoxic agents via linkage to monoclonal antibodies directed against tumor-associated antigens [14]. Importantly, this approach has been clinically validated with four ADCs currently approved for use in human cancer: brentuximab vedotin, a conjugate of an anti-CD30 antibody with monomethyl auristatin E (MMAE) [15] that is approved for relapsed Hodgkin lymphoma, systemic anaplastic large-cell lymphoma, and most recently for subtypes of cutaneous anaplastic large-cell lymphoma; ado-trastuzumab emtansine (T-DM1), a conjugate of trastuzumab with the maytansinoid DM1 [16], used to treat HER2-positive metastatic breast cancer; and two calicheamicin-bearing conjugates, inotuzumab ozogamicin and gemtuzumab ozogamicin, CD22- and CD33-targeting ADCs approved to treat B-cell precursor acute lymphoblastic leukemia or acute myeloid leukemia, respectively. IMGN529 is an ADC comprised of a humanized anti-CD37 monoclonal antibody linked to DM1, which combines the intrinsic proapototic and effector activities of its antibody component with the potent cytotoxic activity of its payload [17]. High affinity binding of IMGN529 to CD37 followed by its internalization results in the intracellular release and accumulation of DM1, which in turn promotes disruption of microtubule assembly, G2/metaphase arrest, and ultimately apoptosis [18]. In preclinical studies, IMGN529 has shown robust antitumor activity in CD37-positive NHL models [17, 19], thus providing a rationale for its clinical evaluation as targeted therapy for the treatment of B-cell malignancies.

This first-in-human, phase I study of IMGN529 monotherapy was designed to assess the overall safety, pharmacokinetics, and preliminary activity of this novel investigational agent in a dose-finding cohort of patients with relapsed or refractory B-cell NHL.

## Patients and methods

### Study design and participants

In this first-in-human, dose-escalation phase I trial, adult patients with relapsed or refractory NHL for whom standard measures did not exist or were no longer effective were enrolled from one European (Switzerland) and five US cancer center sites. To be eligible, patients had to be aged 18 years or older with a histologically confirmed diagnosis of lymphoma limited to DLBCL, FL, MCL, or MZL. Patients were also required to have received at least one prior anti-CD20 based therapeutic regimen, have a life expectancy of greater than 3 months, an Eastern Cooperative Oncology Group Performance status of 2 or lower, and adequate hematological, renal, and hepatic function. Prior therapies resulting in exclusion included chemotherapy or radiation within 3 weeks of study entry, radioimmunotherapy within 2 months of starting study drug, major surgery within 30 days, prior treatment with a CD37-directed agent, and allogenic stem cell transplantation. The trial was conducted in accordance with US Food and Drug Administration regulations, the International Conference on Harmonisation Guidelines for Good Clinical Practice, and the Declaration of Helsinki. The study was compliant with all relevant Institutional Review Board and Independent Ethics Committee requirements and all patients provided written informed consent for participation. The trial is registered at ClinicalTrials.gov, NCT01534715.

### Study design and drug administration

Patients received IMGN529 intravenously on day 1 of a 21-day cycle. The number of cycles was not fixed and patients received IMGN529 until disease progression, unacceptable toxicities, or withdrawal of consent, whichever came first. A conventional 3 + 3 dose-escalation scheme was used, with the first three patient cohort dosed at 0.1 mg/kg. In the initial low dose cohorts, early-onset neutropenia and febrile neutropenia (within 4 days of dosing) were observed and at the 0.4 mg/kg dose level the use of peri-infusional steroid prophylaxis was mandated. Accordingly, patients received 10 mg dexamethasone intravenously 30–60 min prior to infusion and were provided with oral corticosteroids (8 mg dexamethasone or equivalent) to be taken at home on days 2 and 3. However, at the 1.0 mg/kg plus steroid prophylaxis dosing level, febrile neutropenia and neutropenia were experienced by the first three patients treated; these events displayed a delayed-onset profile (days 12–15). Granulocyte growth factor support was subsequently added as primary prophylaxis in every cycle in the days following IMGN529 infusion. This was administered between days 3–10 of each cycle, with the type and duration of growth factor support based on investigator’s decision as per local practice. The combination of peri-infusional steroids and granulocyte growth factor support allowed dose escalation to proceed.

### Safety evaluation

Safety assessments performed at screening included medical history and physical examination, ECOG performance status, electrocardiogram, blood chemistry and hematology, and serum pregnancy test. Adverse events were graded according to the National Cancer Institute Common Terminology Criteria for Adverse Events version 4.0 and monitored from the time of the first study dose until 28 days after treatment cessation. A DLT was defined as any of the following treatment-related adverse events that occurred during cycle 1: grade 4 neutropenia lasting >5 days despite growth factor support; grade 4 febrile neutropenia or febrile neutropenia as defined by an ANC <500/mm^3^ with a sustained temperature above 38.5 °C (101.3 °F); grade 3 thrombocytopenia with bleeding requiring transfusion; grade 4 thrombocytopenia lasting >5 days or with bleeding and/or requiring platelet transfusion; ≥ grade 2 peripheral neuropathy; ≥ 3 vomiting, nausea, or diarrhea despite optimum supportive care; serum creatine or bilirubin levels ≥3 x ULN; or any grade ≥ 3 non-hematologic toxicity (other than alopecia or grade 3 occurrences of fatigue, allergic hypersensitivity reaction, tumor lysis syndrome, or asymptomatic elevations in biochemistry laboratory values lasting ≤7 days). The maximum tolerated dose (MTD) was defined at as the highest dose at which no more than 1 of six patients experienced a DLT.

### Efficacy evaluation

Tumor response assessment was performed per the International Harmonization Project’s Revised Response Criteria for Malignant Lymphoma [20]. Radiographic tumor evaluation, using CT or PET scans, was performed on all patients within 28 days of the first dose of study treatment and repeat assessments performed every third cycle between days 15–21.

### Pharmacokinetic evaluation

For pharmacokinetic (PK) analyses, plasma IMGN529 concentrations were evaluated using validated enzyme-linked immunosorbent assays with a lower limit of quantitation of 19.9 ng/mL (PRA Health Sciences, Lenexa, KS, USA). Plasma concentrations along with relative actual times were used to calculate the PK parameters listed for cycle 1 using noncompartmental methods.

### Outcomes

The primary objectives were to determine the MTD of IMGN529 administered as a single agent once every three weeks to patients with relapsed or refractory NHL, as well as the recommended dose for further phase 2 studies. Secondary objectives were assessments of safety, tolerability, pharmacokinetics, and preliminary evidence of clinical activity according to standard response criteria. Potential predictive biomarkers for efficacy were evaluated as an exploratory objective.

### Statistical methods

The size of the patient cohort was based on a standard 3 + 3 dose-escalation design and no formal sample size calculation was performed. The safety population consisted of all patients who received at least one dose of IMGN529. The response-evaluable population included all patients who had received at least one dose of study drug and completed at least one post-baseline disease assessment. Descriptive analyses were performed using SAS software (version 9.4) with a cutoff date of November 29, 2016. This trial is registered at ClinicalTrials.gov, number NCT01534715.

## Results

### Patient characteristics

The study enrolled 49 heavily pretreated patients with relapsed or refractory B-cell NHL (median of 3 prior systemic regimens [range, 1–13]), including those who had previously undergone radiation and/or autologous stem cell transplant (Table 1). The population had a median age of 65 years, was primarily male (65%), and consisted of individuals with DLBCL (49%), FL (29%), MCL (14%), or MZL (8%). Twenty-eight patients (57%) received 3 or more cycles of IMGN529 therapy (range 1–16).

**Table 1:** Baseline demographics and disease characteristics

	Patients (*n* = 49)
Age, (years)	65 (27–86)
Sex	
Men	32 (65.3)
Women	17 (34.7)
Histology	
Diffuse large B-cell lymphoma (DLBCL)	24 (49.0)
Follicular lymphoma (FL)	14 (28.6)
Mantle cell lymphoma (MCL)	7 (14.3)
Marginal zone lymphoma (MZL)	4 (8.2)
Disease stage at study entry	
I	2 (4.1)
II	5 (10.2)
III	16 (32.7)
IV	26 (53.1)
Number of prior systemic therapies	
1	5 (10.2)
2	9 (18.4)
≥ 3	35 (71.4)
Median	3 (1–13)
Previous radiotherapy	15 (30.6)
Previous autologous transplant	16 (32.7)

Data are median (range) or n (%)

### Safety and tolerability

As part of escalation, doses of IMGN529 were doubled from 0.1 mg/kg to 0.8 mg/kg before DLTs were observed (Table 2). At 0.8 mg/kg, the first two patients experienced DLTs of grade 4 neutropenia and grade 2 peripheral sensory neuropathy, respectively, prompting dose reduction back to 0.4 mg/kg. Two patients subsequently experienced transient grade 3 febrile neutropenia (in the absence of infection) as a DLT in their first cycle of treatment which resulted in a protocol amendment to include peri-infusional prophylactic steroid administration on days 1–3. This modification reduced the incidence of these early-onset (within 4 days of infusion) myelosuppressive events seen in the initial dosing cohorts. Three patients each were then treated at 0.4 mg/kg and 0.7 mg/kg doses on this corticosteroid regimen with no reported DLTs. Among the first three patients in the 1.0 mg/kg dose group, one DLT of grade 3 febrile neutropenia, and two events of grade 4 neutropenia were observed; these were delayed-onset, beginning 12–15 days post-dosing. Granulocyte growth factor support as primary prophylaxis was then implemented for all patients in all cycles. Three subsequent patients were treated at 1.0 mg/kg and no further reports of neutropenia as DLTs were seen. One of six patients in the 1.4 mg/kg cohort with growth factor support experienced a DLT of grade 4 thrombocytopenia. The 1.8 mg/kg dose was poorly tolerated due to the occurrence of multiple DLTs (Table 2). Based on these findings, the MTD of IMGN529 with growth factor support was determined to be 1.4 mg/kg every 3 weeks. The 0.7 mg/kg dose is currently being investigated in combination with rituximab in patients with relapsed or refractory DLBCL and other forms of B-cell NHLs (NCT02564744).

**Table 2:** Dose-escalation schedule and dose-limiting toxicities

Dose (mg/kg)	Prophylaxis	Evaluable patients (*n* = 48*)	Patients with DLT	Dose-limiting toxicity
0.1	None	3	0	–
0.2	None	8	0	–
0.4	None	5	2	Grade 3 febrile neutropenia
0.8	None	2	2	Grade 4 neutropenia; Grade 2 peripheral sensory neuropathy
0.4	Steroids	3	0	–
0.7	Steroids	3	0	–
1.0	Steroids	3	1	Grade 3 febrile neutropenia
1.0	Steroids + G-CSF	3	0	
1.4	Steroids + G-CSF	6	1	Grade 4 thrombocytopenia
1.8	Steroids + G-CSF	12	2	Grade 3 febrile neutropenia; Grade 3 febrile neutropenia plus grade 4 neutropenia and thrombocytopenia

*One patient (0.2 mg/kg cohort) discontinued from study prior to the safety evaluation performed 21 days following the first administration of IMGN529 and was thus non-evaluable for DLT assessment

Adverse events were reported in all 49 patients; with events of grade 3 or higher seen in 32 individuals (65%). Table 3 lists adverse events of all grades reported in more than 10% of the population and any ≥ grade 3 events occurring in 2 or more subjects. Apart from neutropenia, the most frequent events were primarily ≤ grade 2 and included fatigue, pyrexia, thrombocytopenia, nausea, and diarrhea. Adverse events led to treatment discontinuation in 10 patients (20%): one 0.2 mg/kg patient (grade 2 asthenia), one 0·4 mg/kg patient (grade 3 febrile neutropenia), two 0.8 mg/kg patients (the DLTs of grade 2 peripheral sensory neuropathy and grade 4 neutropenia listed above), one 0.7 mg/kg patient (unrelated cardiac arrest which resulted in a fatal outcome), one 1.0 mg/kg patient (grade 3 tumor lysis syndrome), one 1.4 mg/kg patient (invasive ductal breast carcinoma), one 1.8 mg/kg patient (grade 3 pneumonia *Legionella*), plus two additional cases of grade 2 infusion related reactions (one each in the 0.4 with steroid prophylaxis and 1.8 mg/kg cohorts). Twelve patients experienced a serious adverse event; in 8 individuals (16%) these were considered related to study drug, with grade 3 febrile neutropenia the most frequently observed (5 patients; 10%). No treatment-related deaths occurred during the study.

**Table 3:** Adverse events in the safety population

	All patients (n = 49)		
	Grade 1–2	Grade 3	Grade 4
Neutropenia	2 (4.1%)	6 (12.2%)	10 (20.4%)
Fatigue	17 (34.7%)	2 (4.1%)	0
Pyrexia	18 (36.7%)	0	0
Thrombocytopenia	11 (22.4%)	3 (6.1%)	4 (8.2%)
Nausea	15 (30.6%)	0	0
Diarrhea	9 (18.4%)	2 (4.1%)	0
Asthenia	8 (16.3%)	0	0
Anemia	6 (12.2%)	1 (2.0%)	0
Febrile neutropenia	0	6 (12.2%)	1 (2.0%)
Constipation	7 (14.3%)	0	0
Decreased appetite	7 (14.3%)	0	0
Dyspnea	6 (12.2%)	1 (2.0%)	0
Odema peripheral	6 (12.2%)	1 (2.0%)	0
Increased ALT*	4 (8.2%)	2 (4.1%)	0
Increased AST*	5 (10.2%)	1 (2.0%)	0
Hypokalemia	5 (10.2%)	1 (2.0%)	0
Hyperglycemia	4 (8.2%)	1 (2.0%)	1 (2.0%)
Chills	6 (12.2%)	0	0
Muscular weakness	5 (10.2%)	0	0
Leukopenia	0	4 (8.2%)	0
Decreased platelet count	2 (4.1%)	2 (4.1%)	0
Pneumonia	1 (2.0%)	2 (4.1%)	0

Table contains all adverse events of any grade occurring in >10% of patients in the study population, and any grade 3 or worse event occurring in 2 or more patients

*ALT, alanine aminotransferase; AST, aspartate aminotransferase

### Pharmacokinetics

The mean plasma PK parameters for IMGN529, determined for each dose level on the basis of samples obtained during cycle 1, are summarized in Table 4. Clearance (CL) was substantially higher at the lowest doses relative to that observed at the upper end of the dose range and the mean t½ values ranged from 4.9 h at 0.1 mg/kg to greater than 24 h at doses ≥0.8 mg/kg. These changes are consistent with target-mediated drug disposition (TMDD) at low doses, with saturation of this mechanism occurring at higher doses. When the upper dose range (1.0–1.8 mg/kg) was tested, both AUC_∞_ and C_max_ showed dose-proportionality. In each dose cohort, Vz values were consistent with plasma volume, suggesting that IMGN529 is largely confined to the central compartment. In addition, overall PK parameters were generally consistent between cycles 1 and 3 (data not shown), suggesting that PK did not change upon repeat dosing.

**Table 4:** Pharmacokinetic parameters – Cycle 1

Dose (mg/kg)	N	C_max_ (μg/mL)	AUC_∞_ (h*μg/mL)	t_1/2_ (h)	CL (L/h)	V_z_ (L)
0.1	3	2.3 (0.4)	14.6 (8.8)	4.9 (2.2)	0.8 (0.4)	5.1 (1.2)
0.2	8	3.9 (0.9)	63.7 (29.9)	9.8 (3.1)	0.3 (0.2)	3.8 (1.3)
0.4	8	9.1 (4.7)	238.3 (160.6)	21.6 (22.9)	0.3 (0.2)	4.8 (4.0)
0.7	3	13.8 (2.8)	529.5 (391.8)	20.9 (15.9)	0.2 (0.2)	2.6 (0.0)
0.8	2	20.9 (−)	1174 (−)	39.7 (−)	0.07 (−)	4.0 (−)
1.0	6	24.9 (6.1)	1282 (862.8)	30.3 (20.3)	0.07 (0.02)	2.7 (0.7)
1.4	6	36.0 (7.6)	1822 (975.7)	24.9 (6.6)	0.09 (0.04)	2.9 (0.8)
1.8	12	53.5 (20.0)	2525 (1484)	33.9 (21.5)	0.07 (0.03)	2.8 (0.7)

Data are mean (SD). C_max_ = peak concentration. AUC_∞_ = area under the plasma concentration vs time curve extrapolated to infinity. t_1/2_ = half-life. CL = clearance. V_z_ = volume of distribution at terminal phase

### Efficacy

**Fig. 1:**
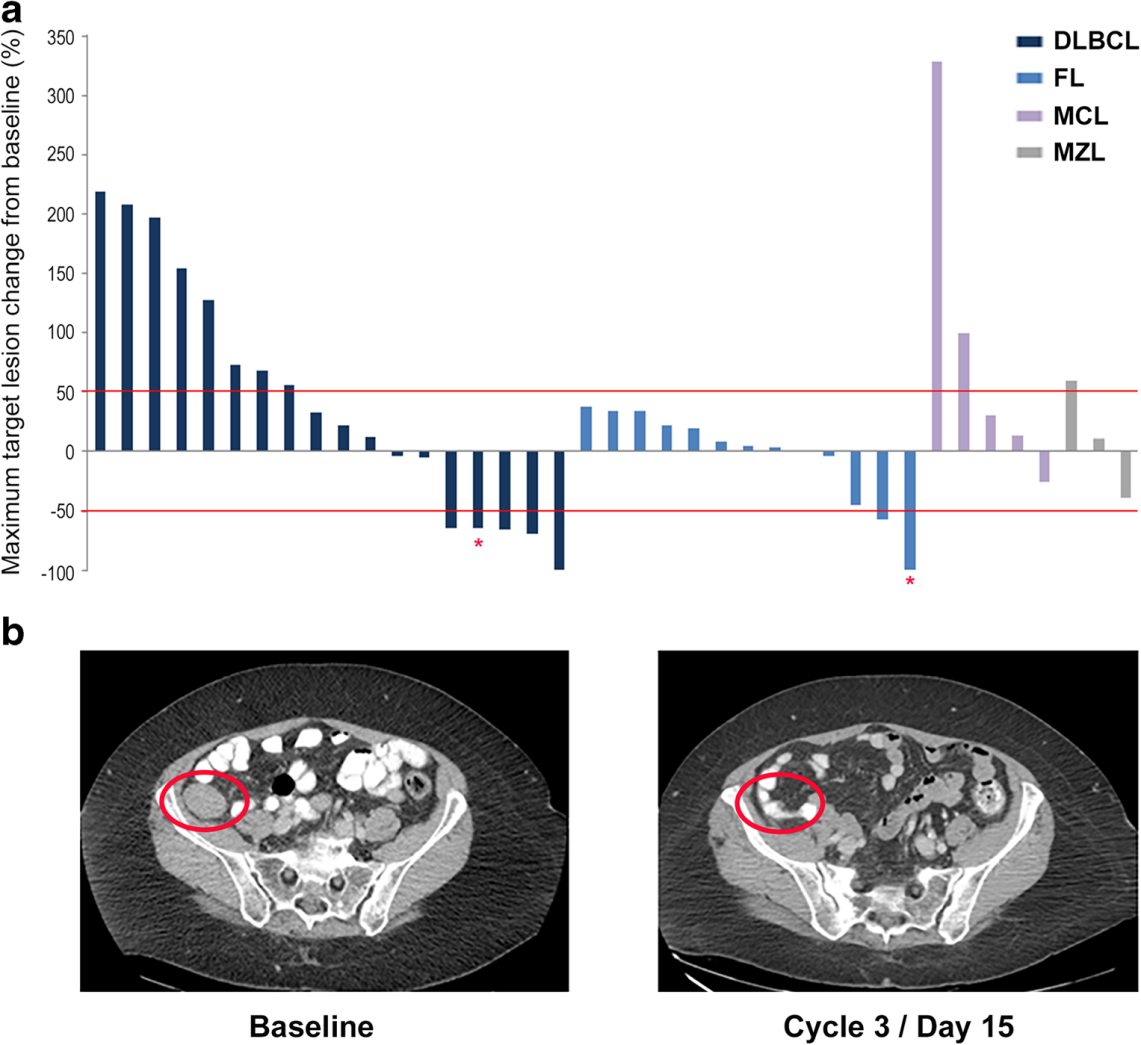
Antitumor responses to IMGN529 monotherapy in patients with B-cell NHL. **a** Maximum changes in target lesions from baseline at best response are shown, measured by CT or PET, according to the Revised Response Criteria for Malignant Lymphoma. Data are for the 39 NHL patients in the response-evaluable dose-escalation population. Each bar represents an individual patient; histological subtypes are grouped as indicated. *Patients had a reduction/disappearance of target lesions, however developed a concurrent new lesion and therefore classified as having progressive disease. **b** Activity of IMGN529 in a relapsed DLBCL patient (non-GCB subtype) who achieved a complete response. The 67-year-old woman had progressed following two earlier lines of chemoimmunotherapy (R-CHOP and R-ICE) and a subsequent autologous stem cell transplant prior to being treated with IMGN529 at 1.0 mg/kg. The red circles show complete regression of the target lesion during the third cycle of treatment

The maximum post-baseline tumor size changes in target lesions for 39 patients who were evaluable for disease response are presented in Fig. 1a. A total of five objective responses were observed, resulting in an overall response rate (ORR) of 13% (Table 5). Four of these (1 complete response [CR] and 3 partial responses [PRs]) occurred in patients with DLBCL, for an ORR of 22% in this lymphoma subset. Notably, the CR occurred in a patient with DLBCL of non-germinal center B-cell (non-GCB) origin treated at 1.0 mg/kg and lasted 4.2 months (Table S1). Pre- and post-treatment scans from this 67-year-old woman, who had previously progressed following two earlier lines of chemoimmunotherapy (R-CHOP and R-ICE) and a subsequent autologous stem cell transplant, are shown in Fig. 1b. Two of the PRs occurred in patients with GCB-DLBCL treated at 0.4 mg/kg and the third in an individual with unclassified DLBCL administered IMGN529 at 1.0 mg/kg, with a duration of response of 8.4 months (Table S1). Another patient with FL accounted for the remaining PR seen on-study. Stable disease was noted in an additional 8 patients (21%) across histological subtypes.

**Table 5:** Best response in evaluable patients (*n* = 39)

	N	Complete response	Partial response	Stable disease	Progressive disease	Overall response rate
DLBCL	18	1 (5.6%)	3 (16.7%)	2 (11.1%)	12 (66.7%)	22.2%
FL	13	0	1 (7.7%)	4 (30.8%)	8 (61.5%)	7.7%
MCL	5	0	0	2 (40.0%)	3 (60.0%)	0
MZL	3	0	0	0	3 (100.0%)	0
Total	39	1 (2.6%)	4 (10.3%)	8 (20.5%)	26 (66.7%)	12.8%

## Discussion

This study established the MTD of IMGN529 as 1.4 mg/kg every 3 weeks, with growth factor support. The dose of 0.7 mg/kg (without growth factor support) every 3 weeks was selected for phase 2 evaluation, with a safety run-in period, in combination with rituximab in patients with relapsed or refractory B-cell NHLs.

The majority of treatment-emergent adverse events, including fatigue, pyrexia, nausea, and diarrhea, were primarily grade 1–2. At low doses (≤ 0.4 mg/kg) without prophylaxis, early-onset grade 3–4 neutropenia and febrile neutropenia emerged as toxicities of note. Peri-infusional steroid use decreased their incidence and permitted further escalation to 1.0 mg/kg. At this level, patients showed delayed-onset febrile neutropenia and neutropenia - which prompted the introduction of granulocyte growth factor prophylaxis. This facilitated neutrophil count recovery and enabled escalation sufficient to declare an MTD. Overall, neutropenia, febrile neutropenia, and thrombocytopenia were the most frequently observed grade ≥ 3 adverse events and the primary DLTs observed. The underlying mechanisms of these myelosuppressive effects are likely linked to off-target effects derived from the microtubule-disrupting function of the DM1 payload in IMGN529. Neutropenia is more consistently observed in ADCs that contain a MMAE payload [21], including brentuximab vedotin [22], and was recently identified as a key toxicity of polatuzumab vedotin, a CD79b-targeting ADC, as part of a dose-escalation study in B-cell NHL patients [23]. Thrombocytopenia has been reported for other DM1-containing conjugates, however is more widespread for calicheamicin-utilizing ADCs [21]. Indeed, the incidence and severity observed in this study compare favorably to those seen in NHL patients treated with inotuzumab ozogamicin, a CD22-targeted ADC containing calicheamicin, where these events were the leading cause of serious adverse events, dose modifications, and discontinuations [24]. The remaining DLT, peripheral neuropathy, was observed in one patient treated at 0.8 mg/kg; however due to an overall low frequency across the safety population, the relevance of this potential toxicity remains undefined.

Pharmacokinetic analysis revealed that IMGN529 exposure did not increase proportionally with dose over the entire dose range during cycle 1. Lower doses (<0.8 mg/kg) showed increased clearance and decreased terminal half-life relative to doses above 1.0 mg/kg, consistent with TMDD [25]. IMGN529 exhibited a dose-proportional increase in AUC_∞_ at doses ≥1.0 mg/kg. Maximal IMGN529 concentrations increased proportionally over the entire dose range, and the volume of distribution was consistent with plasma volume at all doses.

Although only moderate clinical activity was seen across the heterogeneous NHL population, the signals of efficacy observed in patients with DLBCL are encouraging, particularly given that all had advanced disease and were heavily pretreated. While stable disease was most commonly seen in indolent and intermediate types of lymphoma (FL and MCL), the confirmed tumor responses tended to cluster in patients diagnosed with DLBCL. A lack of available tumor tissue precluded complete biomarker analyses and thus no correlations between activity and cell of origin classification or CD37 expression could be determined. However, immunohistochemical detection of CD37 was seen on all evaluable samples. Moreover, it is notable that the patient who achieved a complete response had non-GCB DLBCL, a molecular subtype that confers a poorer prognosis than those of GCB origin [26], as well as high CD37 expression. The duration of response for the additional DLBCL responders lasted as long as 8.4 months, providing further evidence that IMGN529 has meaningful activity in patients with refractory, aggressive disease. Importantly, our findings validate CD37 as a rational target for therapeutic intervention in DLBCL. To date, the initial human evaluations of the CD37-targeting agent TRU-016 have been conducted either as monotherapy in patients with chronic lymphocytic leukemia (CLL) or as part of combinations in indolent lymphomas [27, 28] and therefore the efficacy results seen here cannot be directly compared to those studies. Currently, there is another CD37-targeting ADC in development, AGS67E, which comprises MMAE conjugated to an antibody component that lacks the intrinsic apoptotic and ADCC activity present in IMGN529 [29]. A phase 1 study of AGS67E monotherapy in patients with relapsed/refractory NHL and CLL is ongoing and final results are not yet available; however interim reports have similarly identified objective responses in DLBCL patients as part of that trial [30]. Of note, delayed-onset neutropenia was also identified as a dose-limiting toxicity in the study, which similarly required growth factor support in order to allow escalation to proceed.

Preliminary biomarker analyses were additionally performed using a subset of 33 patient samples that were available for CD37 expression staining, two-thirds of which were found to express the antigen at high levels. Based on the small sample size, however, no statistically relevant relationships between CD37 expression and likelihood of response were detected. Further efforts are therefore needed to identify any predictive or prognostic biomarkers associated with IMGN529 treatment.

Overall, the results of this study support the continued evaluation of IMGN529 as a novel CD37-targeting therapeutic in NHL, based on its manageable safety profile and encouraging signs of clinical efficacy, particularly in patients with DLBCL. In this regard, the clinical exploration of the IMGN529 in combination with rituximab has commenced, with a Phase 2 study in patients with relapsed or refractory B-cell lymphoma currently underway (NCT02564744).

## Electronic supplementary material

ESM 1 (DOCX 26 kb)
