# Supplementary material for: Safety, tolerability, and preliminary activity of IMGN529, a CD37-targeted antibody-drug conjugate, in patients with relapsed or refractory B-cell non-Hodgkin lymphoma: a dose-escalation, phase I study
Source: Invest New Drugs. 2018 Feb 17;36(5):869–76. doi: 10.1007/s10637-018-0570-4 (PMC6153548; doi:10.1007/s10637-018-0570-4)
Supplement: Supplementary file 1 — (DOCX 26 kb) [file 10637_2018_570_MOESM1_ESM.docx]

**Table S1** Disease subtypes and duration of response for patients with an objective tumor response

|  | | |  |  | |  |
| --- | --- | --- | --- | --- | --- | --- |
| Dose (mg/kg) | Best overall response | Lymphoma (grade/subtype) | | | Duration of response (months) | |
| 0.2 | PR | FL (grade 3) | | | 2.0 | |
| 0.4 | PR | DLBCL (GCB) | | | 2.0 | |
| 0.4 | PR | DLBCL (GCB) | | | 1.0 | |
| 1.0 | CR | DLBCL (non-GCB) | | | 4.2 | |
| 1.0 | PR | DLBCL (unclassified) | | | 8.4 | |

CR, complete response; PR, partial response; GCB, germinal center B cell
